# Strong ground motion data of the 2015 Gorkha Nepal earthquake sequence in the Kathmandu Valley

**DOI:** 10.1038/s41597-022-01634-6

**Published:** 2022-08-20

**Authors:** Michiko Shigefuji, Nobuo Takai, Subeg Bijukchhen, Masayoshi Ichiyanagi, Sudhir Rajaure, Megh Raj Dhital, Lalu Prasad Paudel, Tsutomu Sasatani

**Affiliations:** 1 Faculty of Human-Environment Studies, Kyushu University, 744 Motooka, Nishi-ku, Fukuoka, 819-0395 Japan; 2 Faculty of Engineering, Hokkaido University, Kita 13, Nishi 5, Kita-ku, Sapporo, Hokkaido, 060-0808 Japan; 3 Postgraduate Department of Earthquake Engineering, Khwopa Engineering College, Libali, Bhaktapur-8, Bagmati, Nepal; 4 Institute of Seismology and Volcanology, Faculty of Science, Hokkaido University, Kita 10, Nishi 5, Kita-ku, Sapporo, Hokkaido, 060-0810 Japan; 5 Department of Mines and Geology, Lainchaur, Kathmandu, Nepal; 6 Central Department of Geology, Tribhuvan University, Kirtipur, Kathmandu, 44618 Nepal; 7 Tribhuvan University Service Commission, Tribhuvan University, Kirtipur, Kathmandu, 8212 Nepal

**Keywords:** Natural hazards, Seismology

## Abstract

Strong-motion records of earthquakes are used not only to evaluate the source rupture process, seismic wave propagation and strong ground motion characteristics, but also to provide valuable data for earthquake disaster mitigation. The Kathmandu Valley, Nepal, which is characterised by having soft sediments that have been deposited in an earthquake-prone zone, has experienced numerous earthquakes. We have operated four strong-motion stations in the Kathmandu Valley since 2011. These stations recorded the 2015 magnitude 7.8 Gorkha Nepal earthquake that occurred in the Himalayan continental collision zone. For several months after the mainshock, we deployed four additional temporary stations. Here, we describe the seismic data for 18 earthquakes over magnitude 5.0 collected by this array, including the 2015 magnitude 7.3 Dolakha earthquake of maximum aftershock and three large aftershocks of magnitude 6-class. These data are essential for validating the sedimentary structure of the basin and for evaluating the hazard and risk of future earthquakes in the Kathmandu Valley.

## Abstract (summary)

**Table:** 

Measurement(s)	Strong ground motion
Technology Type(s)	Accelerometer
Sample Characteristic - Location	Kathmandu Valley • Nepal

## Background & Summary

The Nepal Himalaya, which formed as a result of the Indian Plate colliding with the Eurasian Plate, underthrusts at a low angle from the Main Frontal Thrust. The Himalayan continental collision zone is one of the most earthquake-prone regions and has experienced several devastating earthquakes in the last millennium (e.g., 1255, 1344, 1408, 1505, 1833, 1934, 2015)^1–3^. The 1934 Nepal-Bihar earthquake, which had a magnitude (*M*) of approximately 8.1, occurred in the Eastern Himalaya region and damaged 19% of buildings in the Kathmandu Valley, more than 100 km away from the epicentre^4,5^. It is considered that great earthquakes could potentially occur in the Central Seismic Gap of the Main Frontal Thrust in the near future^6,7^.

The capital city of Nepal, Kathmandu, is located in the Kathmandu Valley. The oval intermontane valley is approximately 30 km long in the east-west direction and approximately 25 km wide in the north-south direction. The valley floor is overlain by soft, thick fluvial, fluvio-lacustrine, and fluvio-deltaic sediments of Plio-Pleistocene origin^8^, which are more than 650 m thick in the central part of the valley^9,10^. Consequently, the valley is highly susceptible to the risks associated with site amplification^11^. In addition, the explosive population growth that has occurred in recent years has markedly increased the risk of earthquake damage in the valley^12^.

**Fig. 1:**
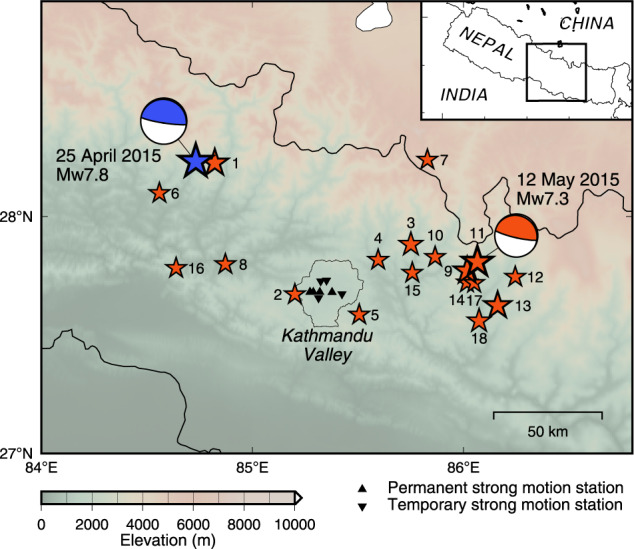
Location map of the epicentres of the 2015 Gorkha earthquake and aftershocks greater than *M* 5.0. The blue and red stars indicate the epicentres for the mainshock and the aftershocks from the USGS-NEIC. The focal mechanisms for the mainshock and the largest aftershock are also shown. Triangles and inverted triangles indicate permanent and temporary stations, respectively. The numbers correspond to Table 2.

To study site amplification in the Kathmandu Valley, Hokkaido University collaborated with Tribhuvan University to install seismic stations for recording strong-motion data in four different parts of the valley, including an exposed rocky site in 2011. On 25 April 2015, the moment magnitude (*M*_w_) 7.8 Gorkha earthquake struck Nepal (Fig. 1). The hypocentral depth was shallow^13^, and the focal area was estimated to be 180 km long and 110 km wide on the low dip-angle fault plane^14^. The rupture propagated to the east, and a large slip area formed near the northern part of the valley^14–16^. This event caused approximately 1,700 deaths, and 13% of the buildings, including World Heritage sites, were damaged inside the valley^17^.

Our array in the valley successfully captured strong ground motion records^18^. During this earthquake, other two seismic observation stations located on sediments in the valley also recorded seismic data: the Kantipath station operated by the United States Geological Survey (USGS)^19^, and the station operated by the Department of Mines and Geology, Ministry of Industry^20^. These data are valuable for evaluating strong ground motion characteristics and clarifying the relationship between strong ground motions and damage. For example, the relationship between the building damage ratios based on the visual damage assessments and fragility curves was discussed^21^.

The mainshock was followed by a series of aftershocks. The ensuing aftershock activities were concentrated in the eastern part of the rupture area^22^. Ten days after the mainshock, four additional stations were installed temporarily along a north-south profile of the valley to investigate the distribution of site amplification in the valley using strong-motion records from the aftershocks. Our array captured the aftershocks^23^, including the largest aftershock (*M*_w_ 7.3) on 12 May 2015 occurred in the Dolakha region^24^ (Fig. 1). The strong ground motion distribution for the mainshock was insufficient to estimate the site amplification and wave propagation characteristics for the entire valley. Data from additional temporary stations and strong-motion records of small and mid-sized earthquakes are useful to estimate the site amplification characteristics and validate the velocity structure models. Ichiyanagi *et al*.^25^ investigated aftershock activity, and Bijukchhen *et al*.^26^ constructed the initial velocity structure model for the Kathmandu Valley based on previous geological and geophysical exploration results and the strong-motion records. Mori *et al*.^27^ proposed correction terms for the amplification by the sedimentary layers in the ground motion prediction equation.

The data were disseminated through the figshare data repository at 10.6084/m9.figshare.19809052^28^. These will prove to be important fundamental data for earthquake disaster mitigation activities in the Kathmandu Valley. For example, these data can be used to produce seismic hazard maps, building damage prediction maps, etc., as well as seismic design of building structures. In this study, we present observation-based strong ground motion data. We introduce our strong-motion array in the Kathmandu Valley and describe the dataset obtained after the 2015 Gorkha earthquake sequence. We also explain the quality of these data.

## Methods

### Strong-motion station array

**Fig. 2:**
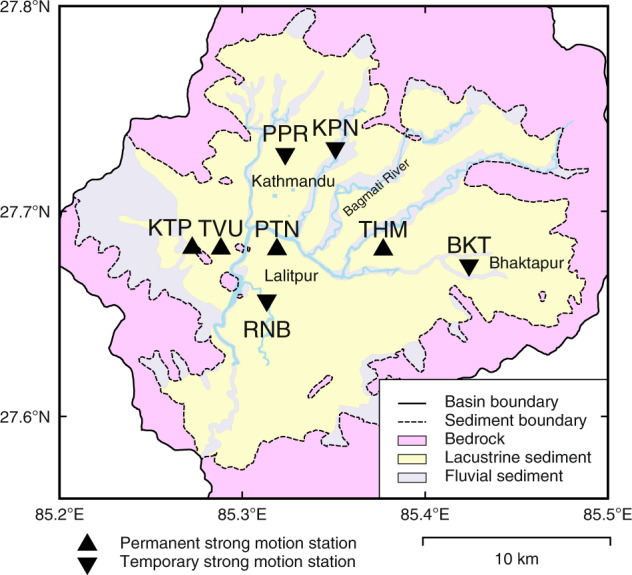
Location of strong-motion stations plotted on the simplified geological map in the Kathmandu Valley (based on Shrestha *et al*.^29^). Triangles indicate permanent stations and inverted triangles are temporary stations.

On 20 September 2011, Hokkaido University and Tribhuvan University installed continuous recording accelerometers along an approximately 10-km east-west axis in four different parts of the valley: stations KTP (Kirtipur municipality office, Kirtipur), TVU (Central Department of Geology, Tribhuvan University, Kirtipur), PTN (Pulchowk Campus, Institute of Engineering, Tribhuvan University, Lalitpur), and THM (University Grants Commission Office, Sanothimi, Bhaktapur). Ten days after the 25 April 2015 Gorkha earthquake, three stations were added along an approximately 8-km north-south axis in the valley: stations RNB (Ranibu, Lalitpur), PPR (Panipokhari, Kathmandu), KPN (Kapan, Kathmandu), and one station was added at Bhaktapur (BKT), a city that suffered heavy damage during the earthquake. A total of eight stations distributed within a radius of approximately 7 km were operated from 5 May 2015 to August 2015. Figure 2 and Table 1 show the locations of the strong-motion arrays. The geological conditions^29^ differed at each of the stations; for example, the KTP site was located on exposed rocky hillocks that breached through the sediments, while the TVU, PTN, THM, BKT, RNB, PPR sites were all located on lake sediments, and the KPN site was located on fluvial sediments of the Bagmati River.

**Table 1:** List of the strong-motion stations.

Code	Site location	Geology	Building type	Installation period
KTP	Kirtipur	Bedrock	Four-story bldg.	September 2011 – present
TVU	Kirtipur	Lacustrine sediment	Two-story bldg.	
PTN	Pulchwok, Lalitpur	Lacustrine sediment	Single-story bldg.	
THM	Sanothimi, Bhaktapur	Lacustrine sediment	Three-story bldg.	
BKT	Bhaktapur	Lacustrine sediment	Four-story bldg.	8 May 2015–6 August 2015
RNB	Ranibu, Lalitpur	Lacustrine sediment	Ground surface	
PPR	Panipokhari, Kathmandu	Lacustrine sediment	Ground surface	
KPN	Kapan, Kathmandu	Fluvial sediment	Single-story bldg.	

### Observation system

The observation equipment was selected to obtain stable records, even under conditions of unstable power supply in Kathmandu. The installed instruments consisted of a highly damped moving coil-type three-component accelerometer (JEP-6A3-2, Mitutoyo Corp., Japan) which does not require a power supply, and a 24 Analog-to-Digital bit low-power data logger (DATAMARK LS-8800, Hakusan Corp., Japan) with an external GPS antenna for time calibration. The sampling rate was 100 Hz and data were recorded continuously at all stations. The accelerometer had a flat response (−3 dB) for ground acceleration from 0.1 Hz to an aliasing frequency with a sensitivity of 0.22 V/m s^−2^. The data loggers at the permanent stations were powered by a DC supply and fitted with a 12 V rechargeable car battery along with a voltage stabiliser. The data loggers at the temporary stations were powered by a 12 V car battery. Due to safety considerations and the high population density in the study area, these instruments were installed on the foundation-level floor of reinforced concrete buildings that were one to four stories high, and outside buildings for a few temporary stations. The GPS antennas were either positioned outside of the buildings or at windows so that they could receive satellite signals. The permanent accelerometers were affixed to the floor with bolts, while temporary accelerometers were affixed using two-part epoxy adhesives. Accelerometers were levelled and oriented along the building or to the magnetic north. Recorded data were stored on 16 GB SD cards and data were collected on-site. Maintenance of the permanent stations was performed at six-month intervals. Permanent stations were maintained two weeks before the 2015 Gorkha earthquake.

### Strong ground motion

**Fig. 3:**
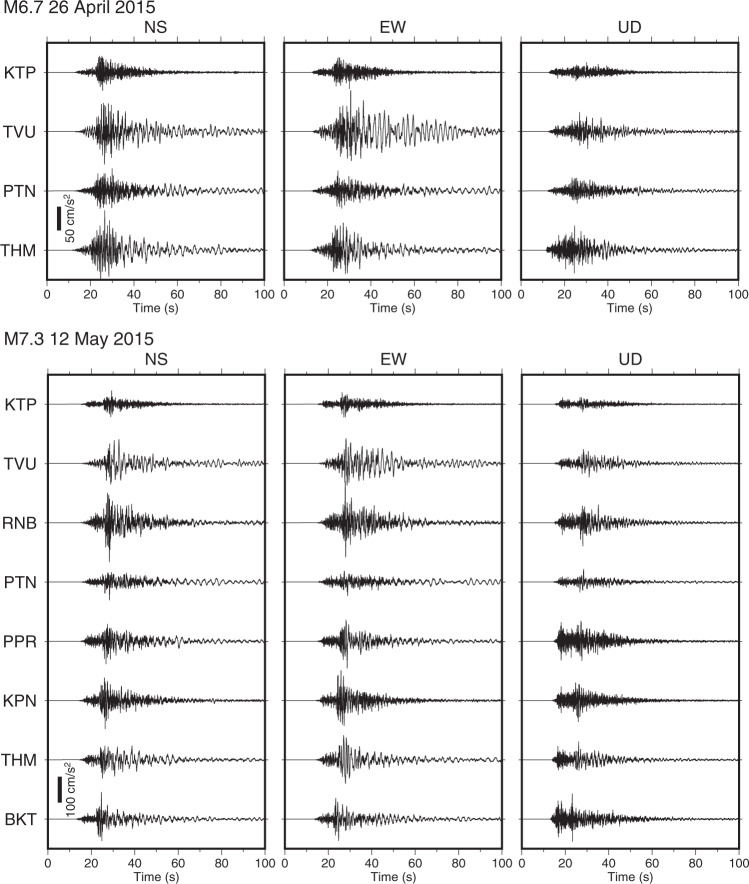
Recorded acceleration waveforms during two earthquakes. Acceleration waveforms for the *M*_w_ 6.7 26 April 2015 earthquake (top) and the 2015 *M*_w_ 7.3 Dolakha earthquake (bottom).

The strong-motion stations recorded the 2015 Gorkha Nepal earthquake sequence data. Figure 1 and Table 2 show 18 mid-to-large sized earthquakes (5.0 < *M* < 7.3) with the strong-motion data of signal-to-noise ratios > 2 located within the epicentral area. Source parameters were from the USGS National Earthquake Information Center (NEIC: https://www.usgs.gov/programs/earthquake-hazards/earthquakes). As examples, Fig. 3 shows the acceleration waveforms of the *M*_w_ 6.7 earthquake (Table 2, No. 9) and the *M*_w_ 7.3 maximum aftershock (Table 2, No. 11). The mainshock records have already been published as supplementary data by Takai *et al*.^18^.

**Table 2:** Source parameters for earthquakes from the USGS-NEIC.

No.	Origin time (UTC: NST-5:45)	Latitude (°N)	Longitude (°E)	Depth (km)	Magnitude	Region
	2015-04-25 06:11:26	28.231	84.731	8.2	*M*_w_ 7.8	Gorkha
1	2015-04-25 06:45:21	28.224	84.822	10.0	*M*_w_ 6.6	Gorkha
2	2015-04-25 06:53:43	27.672	85.201	10.0	mb 5.0	Kathmandu
3	2015-04-25 06:56:34	27.882	85.751	10.0	mb 5.5	Sindhupalchowk
4	2015-04-25 07:47:01	27.817	85.596	10.0	mb 5.0	Sindhupalchowk
5	2015-04-25 08:55:56	27.587	85.506	10.0	mb 5.3	Kavrepalanchok
6	2015-04-25 12:44:05	28.098	84.559	10.0	mb 5.2	Gorkha
7	2015-04-25 17:42:51	28.238	85.829	10.0	*M*_w_ 5.1	Tibet
8	2015-04-25 23:16:15	27.799	84.872	13.6	*M*_w_ 5.1	Dhading
9	2015-04-26 07:09:11	27.771	86.017	22.9	*M*_w_ 6.7	Sindhupalchowk
10	2015-04-26 16:26:07	27.830	85.865	14.0	*M*_w_ 5.0	Sindhupalchowk
11	2015-05-12 07:05:20	27.809	86.066	15.0	*M*_w_ 7.3	Dolakha
12	2015-05-12 07:34:23	27.746	86.245	10.0	mb 5.4	Dolakha
13	2015-05-12 07:36:54	27.625	86.162	15.0	*M*_w_ 6.3	Dolakha
14	2015-05-12 08:06:06	27.722	86.016	15.0	mb 5.0	Dolakha
15	2015-05-12 08:13:55	27.763	85.757	15.0	mb 5.1	Sindhupalchowk
16	2015-05-12 21:25:12	27.783	84.638	10.0	mb 5.2	Dhading
17	2015-05-13 21:38:06	27.720	86.050	8.4	mb 5.0	Dolakha
18	2015-05-16 11:34:10	27.560	86.073	7.0	*M*_w_ 5.5	Dolakha

Events No. 1–10 were recorded by four permanent stations, and events No. 11–18 were observed by eight stations.

## Data Records

The data files as shown in Table 2 were deposited in the figshare data repository at 10.6084/m9.figshare.19809052^28^. Minimum processing included converting the data from raw WIN system^30^ format to acceleration waveform data and correcting the orientation. The data files consist of four columns for time and geographic north-south (NS), east-west (EW), up-down (UD) data in ASCII text format. A recording length is set to 180 s before and after the P- and S-waves. Table 3 shows the data format characteristics. The site location and recording start time (UTC) are described in the file header. The file name consists of a code, earthquake date, and file extension (.txt).

**Table 3:** Format of the published data file.

Line	Contents
1^st^	Array name
2^nd^	Station code name
3^rd^	Station latitude in degrees
4^th^	Station longitude in degrees
5^th^	Station altitude in meter
6^th^	Sampling frequency in Hz
7^th^	Duration time in seconds
8^th^	Recording start time (UTC)
9^th^	Creation date
10^th^	Description of each column (Time, NS, EW, UD)
After 11^th^	Time from recording start time in s, Acceleration waveforms in cm/s^2^

Since the JEP-6A3 accelerometer is an over-damped moving coil mechanical seismograph, it is possible to derive accurate long-period ground motions at <0.1 Hz by correcting for the sensor response. The pendulum motion is proportional to the ground velocity, *h* is a damping constant of 26, and the natural frequency *f*_0_ is 3 Hz. The frequency response function *Λ*(*ω*) has an amplitude of 1 and can be estimated as follows:

1 $${\Lambda }\left(\omega \right)=\frac{{\omega }^{2}}{{\omega }^{2}-{\omega }_{0}^{2}-2h\omega {\omega }_{0}i}\frac{1}{\omega i}$$

where *ω* is the angular frequency and *ω*_0_ is the resonance angular frequency of the pendulum. Therefore, the accelerometer had a flat response for ground acceleration over 0.1 Hz.

## Technical Validation

After the 25 April 2015 Gorkha earthquake, we checked the permanent strong-motion stations. The instruments were undamaged and the buildings in which the equipment were installed were visually assessed as being either undamaged or only slightly damaged^21^. In addition, continuous recordings without any missing data were obtained throughout the event. However, since Kathmandu is a populous city with numerous buildings and heavy traffic, these sources of artificial ambient noise may have contaminated the observed seismic records.

### Ambient noise

**Fig. 4:**
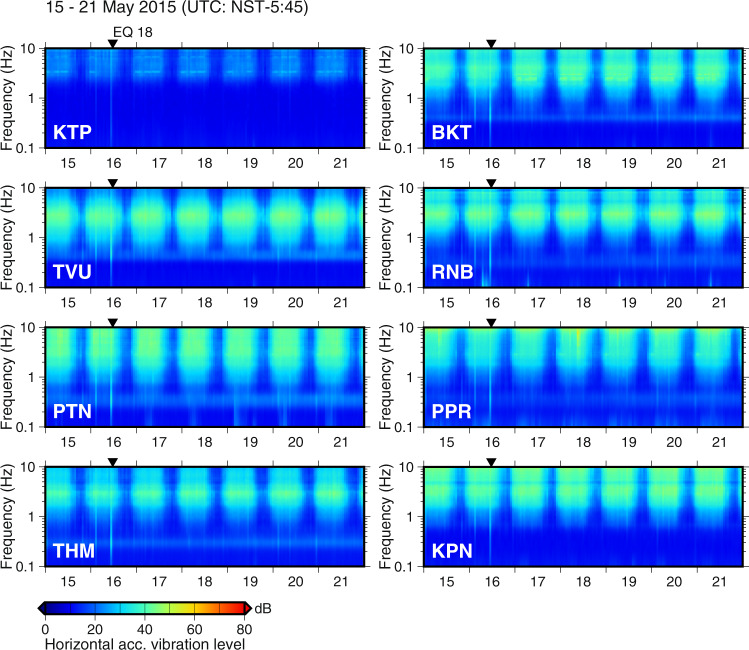
Horizontal acceleration vibration level for microtremors at each station for one week from 15 May 2015 to 21. The time is UTC (NST - 5:45). Inverted triangles indicate the origin time of the earthquake.

In populated metropolitan areas, noise levels attributed to microtremors generated by human activities such as traffic can be very high. Therefore, we estimated the vibration level from the power spectral density (PSD) of acceleration at each site. As an example, the horizontal vibration levels for one week, from 15 to 21 May 2015, are shown in Fig. 4. Observed continuous records were subdivided into intervals of 40.96 s and the PSD of the horizontal component, its root mean square value, and the hourly mean value were calculated. The vibration levels are compared against a reference acceleration vibration level of 10^−6 ^m/s^2^ with no weighting of the vibration sensation. The vibration levels exhibited daily cycles in the high-frequency range; specifically, the difference in the noise level between day and night differed by approximately 20 dB. The microtremors attributed to human activity affect this frequency range, and the vibration levels measured at the sedimentary stations in the central urban areas were larger than those at KTP station on the hilly rock. Conversely, although the amplitudes of the microseisms were small, predominant low-frequency peaks were observed at sedimentary sites.

**Fig. 5:**
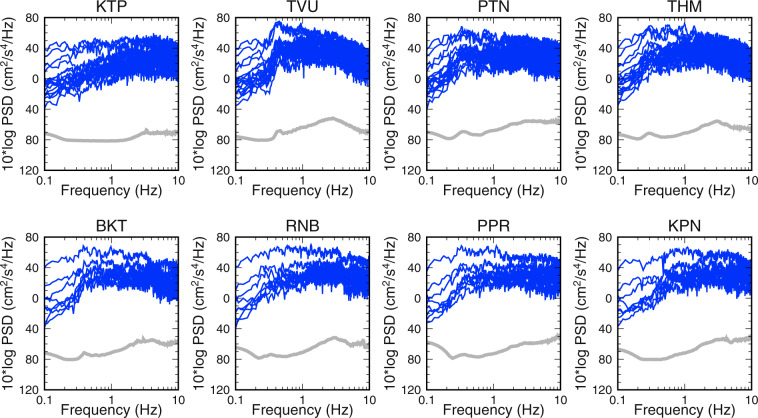
PSDs at each station. Solid grey lines show the average PSDs for microtremors over one day. Solid blue lines show the PSDs for the published earthquake data (5.0 < *M* < 7.3).

Figure 5 shows the average PSD for microtremors not containing seismic data over one day at each station. Compared to the results obtained from the high-noise model^31^, which uses the average value of the high background noise power obtained from broadband seismometers, the PSDs recorded by our array were slightly higher than those estimated by this model. Although the noise levels were relatively high, the PSDs for the recorded earthquake data far exceed those estimated using the model. However, the observed data may contain records from multiple earthquakes because recordings were performed immediately after large earthquakes.

### Sensor response

**Fig. 6:**
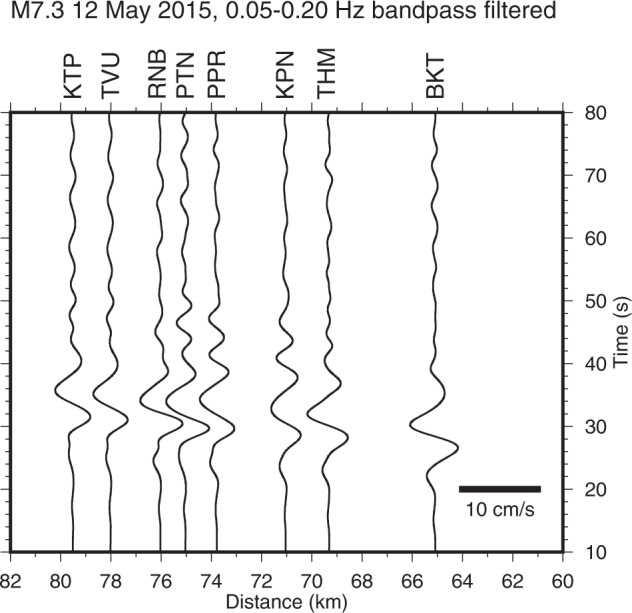
Velocity waveforms for the UD component of the *M*_w_ 7.3 Dolakha earthquake bandpass filtered at 0.05–0.2 Hz.

The 0.05–0.2 Hz bandpass-filtered velocity waveforms for the UD component from the *M*_w_ 7.3 Dolakha earthquake are shown in Fig. 6. We applied a correction of the sensor-response to observed records and derived accurate ground motions even at frequencies of ≤0.1 Hz. The waveforms were coherent in this low-frequency range. It corroborated the findings of Takai *et al*.^18^ who compared the displacement waveforms of the 2015 Gorkha earthquake to the high-rate GNSS waveforms and obtained similar waveforms with static deformation.

## Data Availability

No specific custom code was used to generate the dataset in the manuscript. The data, which are provided in ASCII text format, can be freely downloaded from the figshare data repository and analysed using any software. We used Generic Mapping Tools^32^ to compile several figures.
